# Diagnostic Process of an Ancient Colonnade Using 3D High-Resolution Models with Non-Invasive Multi Techniques

**DOI:** 10.3390/s23063098

**Published:** 2023-03-14

**Authors:** Giuseppe Casula, Silvana Fais, Francesco Cuccuru, Maria Giovanna Bianchi, Paola Ligas

**Affiliations:** 1 Istituto Nazionale di Geofisica e Vulcanologia (INGV)—Sezione di Bologna, Viale Berti Pichat 6/2, 40127 Bologna, Italy; mariagiovanna.bianchi@ingv.it; 2 Department of Environmental Civil Engineering and Architecture (DICAAR), University of Cagliari, Via Marengo 2, 09123 Cagliari, Italy; sfais@unica.it (S.F.); cuccuru.f@gmail.com (F.C.); pligas@unica.it (P.L.); 3 Consorzio Interuniversitario Nazionale per l’Ingegneria delle Georisorse, CINIGEO, Palazzo Baleani, Corso Vittorio Emanuele II 244, 00186 Roma, Italy; 4 National Research Council of Italy (CNR)—Institute of Environmental Geology and Geoengineering (IGAG), Via Marengo 2, 09123 Cagliari, Italy

**Keywords:** close-range photogrammetry, terrestrial laser scanner, 3D digital models, 3D ultrasonic tomography, petrographic analyses, carbonate materials

## Abstract

Here, an avant-garde study of three ancient Doric columns of the precious, ancient Romanesque church of Saints Lorenzo and Pancrazio in the historical town center of Cagliari (Italy) is presented based on the integrated application of different non-destructive testing methods. The limitations of each methodology are overcome by the synergistic application of these methods, affording an accurate, complete 3D image of the studied elements. Our procedure begins with a macroscopic in situ analysis to provide a preliminary diagnosis of the conditions of the building materials. The next step is laboratory tests, in which the porosity and other textural characteristics of the carbonate building materials are studied by optical and scanning electron microscopy. After this, a survey with a terrestrial laser scanner and close-range photogrammetry is planned and executed to produce accurate high-resolution 3D digital models of the entire church and the ancient columns inside. This was the main objective of this study. The high-resolution 3D models allowed us to identify architectural complications occurring in historical buildings. The 3D reconstruction with the above metric techniques was indispensable for planning and carrying out the 3D ultrasonic tomography, which played an important role in detecting defects, voids, and flaws within the body of the studied columns by analyzing the propagation of the ultrasonic waves. The high-resolution 3D multiparametric models allowed us to obtain an extremely accurate picture of the conservation state of the studied columns in order to locate and characterize both shallow and internal defects in the building materials. This integrated procedure can aid in the control of the spatial and temporal variations in the materials’ properties and provides information on the process of deterioration in order to allow adequate restoration solutions to be developed and the structural health of the artefact to be monitored.

## 1. Introduction

A fundamental step in achieving the difficult goal of detecting the state of conservation of the shallow and deeper zones of the stone materials that make up monuments is the integrated use of different non-destructive testing (NDT) methods, such as the terrestrial laser scanner (TLS), close-range photogrammetry (CRP) based on the structure from motion (SfM), and ultrasonic tomography. This is complemented by the study of the petrographic and petrophysical characteristics of the investigated stone materials. In fact, having thorough knowledge of the processes at the basis of the decay of stone materials together with their alteration mechanisms depends mainly on knowing the intrinsic properties of the material composing the artifacts of historical buildings.

Nowadays, digital photogrammetry techniques, such as CRP and TLS, are among the most widely used methodologies to monitor historic and heritage buildings. Thanks to their high productivity over a short time period, they are capable of offering high-resolution 3D models [1,2,3]. This digital documentation provides the basis for the management and maintenance of monumental structures over time and provides information that is suitable for reaching different targets, as well as providing useful data for the different branches of applied research [4,5,6]. In particular, CRP and TLS are very useful for the analysis of shallow parts of stone building materials, as they allow the inspection of large surfaces without any contact or damage [7,8,9,10,11]. However, during a diagnostic process on the conservation state of stone building materials, even when these technologies are applied in synergy, they need to be complemented by the parallel application of other diagnostic techniques, such as ultrasonic techniques that inspect the materials inside based on an analysis of the propagation of the ultrasonic signals [12,13,14,15,16,17,18,19,20,21,22,23]. The ultrasonic velocity distribution is strictly related to the physical condition of the rocks [20,24,25] and to many petrophysical properties [26,27]. This relationship is different for different types of rocks and strictly depends on the rock-forming minerals, chemical composition, type of porosity, density, elastic moduli, fracturing, and weathering [6,28,29,30,31,32].

A 3D data volume can be produced for each investigated physical parameter, and their integrated interpretation represents the final step of the different independent processes. In this way, the final high-resolution 3D models produced can provide complete 3D mapping of the studied artifacts, which is useful for conservation and restoration [2,3,33,34,35,36].

The TLS is a contactless, active, proximal sensing technique in which a laser beam in the 1–1.5 nm frequency (mainly in the near-infrared band) is deflected over a calibrated angular grid, providing automatic coverage of the surfaces of the targeted studied objects, in a field of view that can be suitably adjusted by the operators [3,14,17]. The modern TLSs used in architecture are mainly phase-shift scanners that can acquire millions of pixels per second over a range of distances, generally between 80 and 350 m, and over a field of view of between 320 and 360 degrees [37,38].

CRP is a contactless, active proximal sensing technique in which a digital camera is used to acquire good quality 2D images around the studied object. Using SfM technology, high-density point clouds containing millions of points can be generated, forming 3D models of the studied objects texturized with natural colours derived from the input images [39,40,41].

Moreover, when CRP is applied to non-collaborative surfaces that are featureless or display repetitive patterns, the synergistic application of the photogrammetric method with the photometric stereo to determine the surface orientation from multiple images is effective for overcoming the individual limits of these two techniques and reconstructing an accurate and high-resolution topography [42]. The accuracy of the resulting 3D models is better than 0.1 mm, especially when also using lighting estimation and relighting for photometric stereo (LERPS) and Incorporating Lambertian priors into surface normal measurements as complementary methods [43,44].

The problem with CRP-derived 3D models that are based on SfM technology is their lack of scale and distance, but if a TLS survey is available, the scale factor of the photogrammetric models can be improved by undergoing adjustment in a least square sense using iterative closest point (ICP) styled methodologies [45,46].

For these reasons, we performed a synergistic application of TLS and CRP to produce high-resolution 3D models of complex-shaped bodies texturized with natural colours on a RGB scale together with the reflectivity of the TLS and the correct scale and coordinates.

The 3D ultrasonic travel-times tomography complemented by petrographical and petrophysical analyses based on optical microscopy (OM), scanning electron microscopy (SEM), and mercury intrusion porosimetry (MIP) represents a high-resolution technique to inspect the inner parts of investigated structures. This technique provides information on the elastic properties of the materials related to petrophysical and mechanical properties and other factors, such as the presence of fractures, voids, and flaws. In fact, the velocity propagation of the ultrasonic signals inside the materials decreases as the defects, fractures, or voids increase. Often the factors affecting the velocity propagation of the ultrasonic signal inside the material interact, so the variation of one factor could influence many others. For this reason, knowledge of the textural characteristics of the investigated material is of paramount importance for the reliable interpretation of the ultrasonic signal propagation [16,17]. In this paper, we carried out an in situ ultrasonic survey at a resonance frequency of 24 kHz by acquiring and processing 3D ultrasonic tomography data (namely P-wave travel-times). The use of the longitudinal ultrasonic wave (P-wave) rather than the transversal wave (S-wave) to determine the velocity propagation of the signal is more appropriate when determining the propagation velocity in an in situ inspection, since the first arrival of the ultrasonic signal can be interpreted unambiguously as a compressional wave along the shortest accessible acoustic path.

In planning and interpreting the 3D ultrasonic tomography, the CRP and TLS techniques played a fundamental role, since they provided high-resolution 3D digital models on which to carry out a large quantity of measurements (e.g., precise locations of the signal transmission and receiver points). This contributes to the best ultrasonic tomography results in terms of the accuracy and thus the precise locations of longitudinal velocity changes in relation to the textural variation of the materials and the presence of fractures or damage inside them.

**Figure 1:**
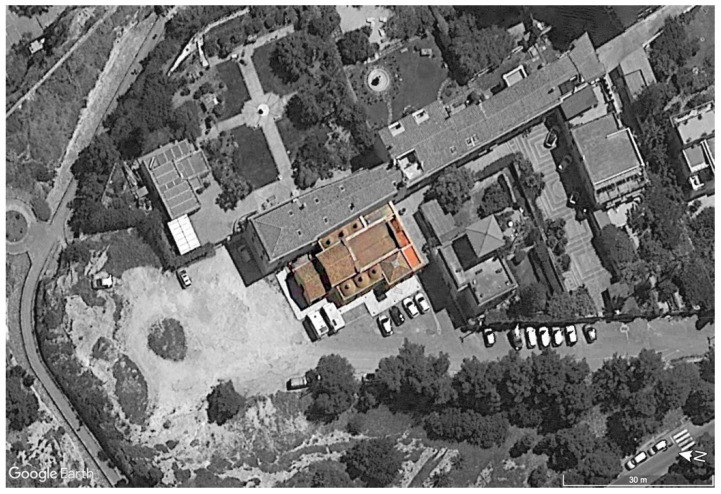
Aerial view of the ancient Romanesque church of Saints Lorenzo and Pancrazio, which dates from the second half of the 13th century and is located in the historical town of Cagliari (Italy) (elaborated from Google Earth).

**Figure 2:**
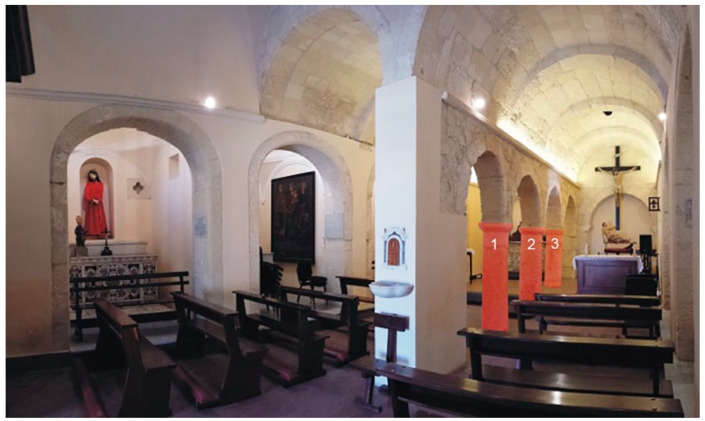
The three studied columns inside the church are highlighted in red and numbered as column 1, column 2, and column 3.

This work describes the integrated application of the diagnostic methods presented above for the study of the ancient Romanesque church of Saints Lorenzo and Pancrazio (Figure 1), which dates from the second half of the 13th century and is located in the historical part of Cagliari (Italy). The analysis focused especially on the three carbonate supporting columns (Figure 2) inside the church.

The workflow is organized as follows: In the Section 2, we present the material characteristics deduced by the OM and SEM analyses and describe the data acquisition with the NDT methodologies and data elaboration strategies. In the following section, the results from the various applied techniques are presented and compared, and finally, these results are also discussed based on the petrographical characteristics of the materials of the studied artifacts.

## 2. Materials and Methods

As previously described, an application of a non-invasive diagnostic methodology based on the integration of visual inspection, Close-Range Photogrammetry (CRP), Terrestrial Laser Scanner (TLS), and low frequency 3D ultrasonic tomography methods was implemented and applied to provide reliable 3D results that can be analyzed and used for both the restoration and conservation of investigated heritage structures. The above methods were supported by optical and electron microscopy data and mercury porosimetry to provide in depth knowledge on the investigated materials.

### 2.1. Materials

The Saints Lorenzo and Pancrazio church was built from local stone ashlars made up of the local Miocene carbonate lithologies known as Calcari di Cagliari Auct. [47,48,49]. The Calcari di Cagliari can be grouped in three different facies known, from bottom to top, as Pietra Cantone, Tramezzario, and Pietra Forte, according to their different textural and petrophysical characteristics.

The Pietra Cantone is a yellow bioclastic, mud-supported, poorly stratified, and intensely bioturbated limestone that dates back to the Tortonian [48]. This stone is characterized by a high content of planktonic and benthic foraminifera and small amounts of iron oxides and terrigenous components, such as quartz, feldspars, and biotite. The depositional environment indicates a paleobathimetry of about 60–80 m, referable to the circalittoral plane [50].

The Tramezzario is a white bioclastic grain-supported limestone that dates back to the Tortonian [51]. This lithotype is usually well lithified, without stratification, and characterized by a rich fossiliferous content that mainly includes bivalves, echinoids, bryozoans, crustaceans, and algal rhodolites, bounded by sparry calcite cement. The biocenosis indicates a paleobathymetry of less than 40 m, referable to the infralittoral—circalittoral plane [50].

The Pietra Forte is a creamy white massive shelf limestone with yellow and red specks that dates back to the Tortonian—Messinian [50,51]. The fossiliferous content, typical of a littoral environment with a paleobathymetry of less than 30 m [50], is mainly characterised by Lithothamnium algae build-up and associations of bivalves, gastropods, and bryozoans.

**Figure 3:**
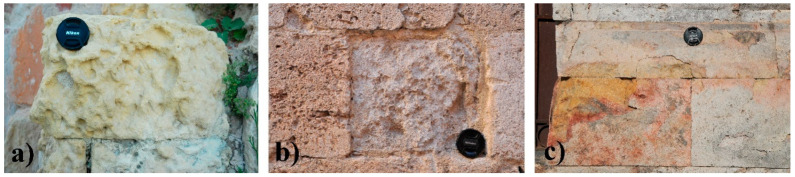
Degradation forms of the Calcari di Cagliari used as building materials: (**a**) Alveolization of an ashlar of Pietra Cantone; (**b**) loss of material due to pulverization and detachment in an ashlar of Tramezzario; (**c**) fractures, oxidation, and detachments in a wall built with Pietra Forte ashlars.

The different textural and petrophysical properties of the three lithotypes influence the forms and intensity of the degradation [52,53,54]. The Pietra Cantone is a very soft stone due to its high content of carbonate mud. The degree of poor cementation of granules causes significant degradation forms, mostly when the rock is water-saturated. This condition, combined with the presence of salts, causes alveolization and pulverization on both the outcrops and the monument built with this lithotype (Figure 3a). 

The Tramezzario is usually a hard, compact stone, although in several cases, it can be characterized by the combination of weak cementation and high secondary porosity values that contribute to the development of severe degradation forms such as detachment and pulverization phenomena (Figure 3b). 

The Pietra Forte is generally a very compact, tenacious, and hard limestone that feels smooth to the touch after cutting. Due to its excellent cementation, the Pietra Forte is less subject to degradation; however, fractures, oxidation, and detachments are not unusual (Figure 3c). Fractures are often caused by stress that exceeds the rock’s strength and causes a loss of cohesion along its weakest plane, while the detachment and loss of material is generally due to the release of internal tensions.

Due to its peculiarities, each facies of the Calcari di Cagliari was used in the construction of different architectural elements of the church of Saints Lorenzo and Pancrazio. Being easily cut, the Pietra Cantone is the most frequently used building material and was used almost exclusively to form the ashlars of the two barrel vaults and the walls of the church. Tramezzario was partially used in the construction of the walls and can be present in specific architectural elements, such as door lintels. Due to its excellent mechanical characteristics, Pietra Forte was used for the construction of the three investigated supporting columns.

### 2.2. Macroscopic Features

Based on the knowledge of the above materials, we carried out an in situ macroscopic analysis on the three investigated supporting columns, which are the main focus of our work. All columns, hereinafter called from us as column 1, column 2, and column 3, are made from Pietra Forte limestone. They are characterized by a simple capital in Doric style and a stocky stem that rests directly on the church floor.

**Figure 4:**
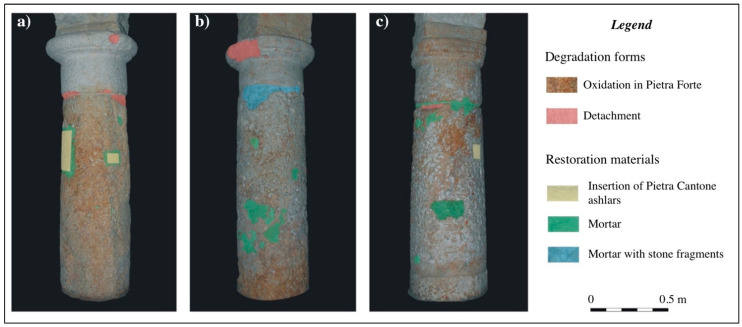
Macroscopic characteristics of the studied columns: (**a**) Column 1; (**b**) Column 2; (**c**) Column 3.

At the preliminary macroscopic observation (Figure 4a–c) the columns were characterized by shallow alteration, such as oxidation, causing the lithotype to be red in several sectors. In different parts of the capitals and in the joint between the stem and the capital, a few material detachments can be seen due to physical causes. Previous restoration works are clearly visible in all three columns. The most common form of restoration is mortar applications, sometimes combined with stone fragments of Pietra Cantone and Tramezzario as filling materials (Figure 4b). In columns 1 and 3, restoration works characterized by prismatic additions of Pietra Cantone are clearly visible (Figure 4a–c). Additions of such a lithotype with very different petrophysical and textural properties to the column building material (Pietra Forte) may not be appropriate and could even be harmful, especially if they develop deep into the inner parts of the columns.

### 2.3. Optical and Scanning Electron Microscopy

Thin sections of the above carbonate materials were analyzed by optical microscopy (OM) and scanning electron microscopy (SEM) in order to identify their textural characteristics and porosity. The porosity was also examined by mercury intrusion porosimetry (MIP). The OM analysis was carried out by means of a petrographic microscope in polarized light (Carl Zeiss Axioplan microscope—Carl Zeiss, Oberkochen, Germany). The SEM analysis was carried out by a Zeiss EVO 50 VP model scanning electron microscope (Carl Zeiss, Oberkochen, Germany) connected to an EDS X-Max (Oxford Analytical Instruments Ltd., High Wycombe, UK). SEM analyses were performed under a high vacuum condition with an Electron High Tension (EHT) of 20 kV, a Working Distance (WD) of 10 mm, an Iprobe current of 200 pA, and a resolution of about 1–2 nm. The thin sections studied in OM were treated with blue dye epoxy resin to improve the identification of the porous system [16,17,25,26,55], while for the SEM analyses, the thin sections were metal-coated, creating a conductive 10 nm layer of gold on their surfaces to inhibit charging, reduce thermal damage, and improve the secondary electron signal required for the morphology examination. The evaluation of the porosity carried out with OM and SEM was accomplished by MIP, which provides a measure of connected porosity. A Micromeritics Autopore IV 9500 (Micromeritics Instrument Corporation, Norcross, GA, USA) was used to measure the connected porosity in fifteen representative samples of the Calcari di Cagliari.

### 2.4. Terrestrial Laser Scanner

As described above, during a TLS survey, the radiation transmitted from the laser beam must be able to reach the scatterers on the surface of the studied targeted object and then return to the laser sensor (two-way travel), where it is received and sensed. When a phase shift scanner is operated, a phase comparator integrated inside the TLS sensor detects the phase shift between emitted signal and received backscattered signal that is proportional to the beam’s incidence angle and to the distance between the laser sensor and the illuminated points. During a TLS survey, the acquired data are several point clouds (at least one for every station point) containing the point positions in an arbitrary reference frame (a point inside the laser sensor) and the intensity parameter mainly on a 0–255 (or 0–1) scale that represents, in practice, the power of the backscattered radiation. 

**Figure 5:**
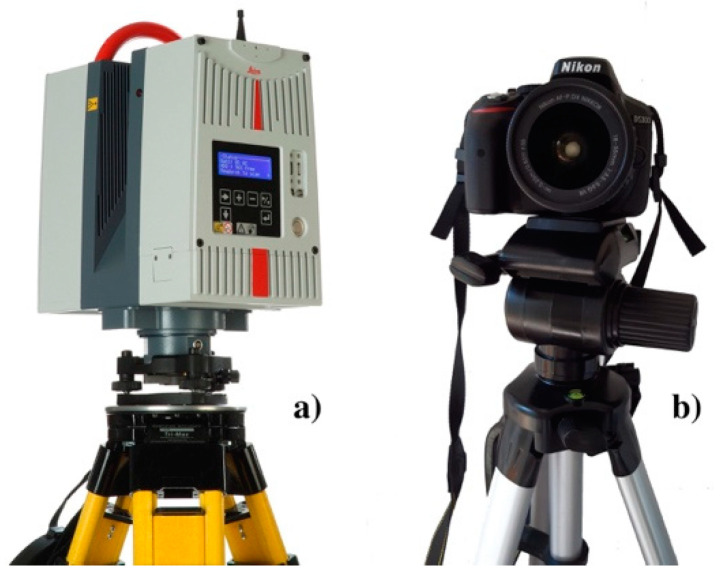
(**a**) The Leica HDS-6200 TLS; (**b**) The Nikon D5300 digital reflex camera.

In this work, we performed a TLS survey by installing a Leica HDS-6200 (Figure 5a) phase-shift scanner on about 60 survey stations distributed along the plan and inside the church of Saints Lorenzo and Pancrazio, covering the entire perimeter. The resulting filtered, registered, aggregated, unstructured point cloud consisted of about 1.3 Tb of points. We used only twelve (four for every studied column) of the original sixty point clouds to aggregate the 3D models of the studied columns. These twelve point clouds were acquired at fixed distances and with laser beam incidence angles in the range of 0–30°, all along the studied columns using the highest scanner resolution. During the TLS survey, the scanner was installed on a levelled geodetic tripod.

#### TLS Data Processing

**Figure 6:**
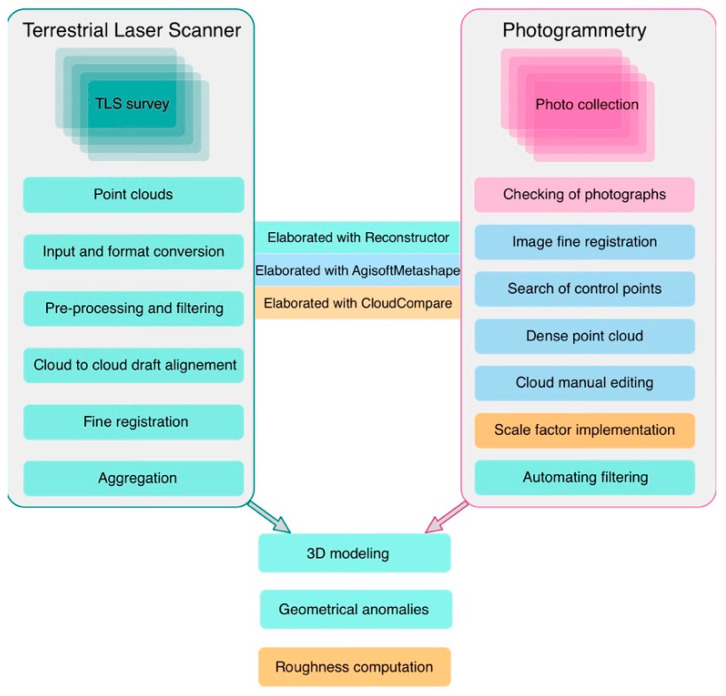
Flow chart representing the TLS and CRP data processes adopted in this work to compute high-resolution 3D models of the entire church and, subsequently, the three studied columns. The operations performed with JRC-3D Reconstructor^®^ version 4.1.2, Agistoft Metashape^®^ version 1.8.4, and CloudCompare version 2.12.4 software are highlighted in the figure with green, blue, and orange rectangles, respectively.

The TLS data processing followed the scheme presented in Figure 6. We used the JRC 3D Reconstructor^®^ software version 4.1.2 package by Gexcel^®^ to process the TLS data. This began with point cloud importation, followed by manual editing and automatic filtering of the point cloud to remove the unwanted noisy data, and then point cloud to cloud draft automatic data registration. In some cases, we had to use manual preregistration. The procedure was followed by a first automatic fine registration with iterative closest point (ICP) algorithms suitably implemented in the Reconstructor^®^. This operation was performed with a standard deviation of a few centimetres (0.15 m), while the final step of the registration process was completed with the aid of bundle adjustment algorithms with a final standard deviation of 1 to 2 mm. The aggregation of filtered registered point clouds was then applied with the consequent generation of a dense, high-resolution 3D model texturized with the intensity of the used TLS. The final step of our procedure was 3D model inspection and the computation of geometrical anomalies that needed to be compared with the results of other diagnostic techniques, such as ultrasonic tomography.

### 2.5. Close-Range Photogrammetry

The CRP technique based on SfM is a proximal sensing NDT methodology used for the estimation of high-resolution 3D models from different sequences of overlapping 2D images of a studied object. SfM photogrammetry is commonly used to define the entire reconstruction workflow of artifacts, starting from sets of good quality images to generate a dense point cloud [8,11,41].

In this work, we started the CRP workflow with the acquisition of a set of high-quality color images of the studied object using a Single Lens Reflex Digital Camera (Nikon D-5300) (Figure 5b) equipped with a complementary metal-oxide semiconductor (CMOS) sensor with a resolution of 24.2 Mpx. The camera was mounted on a tripod equipped with spherical levels settled at different equidistant station points around the object of study. The resulting images, which needed to overlap by 60–70%, were processed using Agisoft-Metashape^®^ software based on an SfM-style methodology. In the case of the Saints Lorenzo and Pancrazio church, we used the CRP technique in synergy with TLS. We operated the Nikon digital camera on about 30 station points distributed at regular steps around the three studied columns. 

#### CRP—Data Processing

The CRP data processing procedure is schematized in Figure 6. It began with image input and fine registration, followed by the computation of dense point clouds in a format compatible with the Reconstructor^®^ and CloudCompare input (E57, LIDAR point cloud data format) [56]. The scale factor of the photogrammetry was adjusted with the aid of the TLS 3D model using bundle adjustment algorithms implemented in the CloudCompare software. As a matter of fact, 3D TLS models are characterized by a calibrated scale, distance, and geometry, but they are texturized by the intensity. Conversely, 3D CRP models are lacking in terms of scale and distance, but they are characterized by a higher resolution and are texturized with natural colours. The synergistic application of both of these methods provides an high-resolution 3D model (0.5 mm) that is texturized with both natural colours and reflectivity that can be used more effectively for the diagnostics of materials in comparison with other methods.

### 2.6. Computation of the Geometrical Anomalies

As described above, we used the high-resolution 3D models of the TLS to adjust the scale and coordinates of the high-resolution 3D CRP models. To perform this task, we utilized the bundle adjustment algorithms of CloudCompare software using an iterative procedure based on a least square method.

Subsequently, we used two different techniques to compute the geometrical anomalies of the three columns:

(1) The inspection procedure of Reconstructor^®^ software can be summarized by the following points: (a) A cylinder is fitted in the least square sense to the 3D model of the studied column; (b) a triangular mesh with 1 mm × 1 mm steps is computed from the cylindric geometry together with its cylindric camera; (c) residuals are computed between the 3D model of the column (i.e., aggregated, filtered, and registered unstructured point cloud), and the cylinder taking its triangular mesh as a reference.

The same computation was performed using CloudCompare software. First, a cylinder was generated using the random sample consensus (RANSAC) for a point-cloud shape detection plugin of the software [57]; in the second step, we computed the distances between the registered clouds of the 3D column models and the corresponding cylindric primitive. In fact, the RANSAC styled algorithm plugin can extract shapes from the point data using the smallest number of points required to uniquely define a given type of geometric primitive and generate a corresponding shape. The resulting primitives were tested against all points in the cloud to determine the number of points that were well approximated by the primitive.

(2) The CloudCompare roughness estimation procedure can be described as follows: For each point of the studied cloud, the ‘roughness’ value is defined as being equal to the distance between this point and the best fitting plane computed on its nearest neighbors. When the number of neighboring points is less than three, it is impossible to compute a least square plane. These points are consequently flagged with an invalid scalar value (NaN) and presented in gray.

### 2.7. Ultrasonic Measurements

Based on the 3D models obtained with the previous techniques (CRP and SfM photogrammetry, TLS), 3D ultrasonic tomography was planned by designing a dense, 3D survey to ensure very good spatial coverage of the investigated columns. The 3D CRP and TLS models allowed us to precisely locate the source and receiver stations for the 3D ultrasonic tomography, measure their mutual distance accurately, and detect their coordinates in the selected reference system (Cartesian system). The experimental ultrasonic investigation on the columns was aimed at determining the sizes and locations of potential internal defects and weakness zones. In particular, density and elastic properties represent the material properties that can affect ultrasonic signal propagation, and variations in these properties can be detected from the measurement of the propagation velocity of the ultrasonic signal through the material. 

In turn, the elastic constants and density depend mainly on properties such as the mineralogical content, porosity, tortuosity of the porous system, rock texture, grain size, and shape [26,29,30,32,58]. The delay in transit time of the ultrasonic signal can be the result of a worsening of the elastic characteristics of the material and a decrease in its density caused by textural variations or the presence of cracks, pores, and degradation zones that can change the internal structure of the stone material. 

Ultrasonic measurements on the columns were carried out using a portable ultrasonic non-destructive digital indicating tester (PUNDIT Lab Plus) device (Proceq, Schwerzenbach, Switzerland) interfaced to a portable oscilloscope (Fluke 96B) to acquire and digitalize the ultrasonic waveforms to be analyzed and processed. The experimental device used for the ultrasonic measurements included a set of ultrasonic piezoelectric transducers that could act as either receivers or transmitters and were characterized by a central frequency of 24 kHz for longitudinal waves (P) that propagate through the materials. The 24 kHz central frequency was selected based on preliminary tests and was considered an acceptable compromise between the resolving power and a tolerable attenuation. Based on an analysis of the ultrasonic waveforms, the travel time propagation of the longitudinal ultrasonic signal along many source–receiver paths was measured by locating the first break in the received signal, as observed on the oscilloscope. In this study, only the P wave first arrival for each source–receiver path was taken into account, as this wave is experimentally easy to detect, ensuring a reliable analysis. The measuring range was from 0.1 µs to 9999.9 µs and the accuracy was 0.1 µs.

Considering that the surfaces of the column shafts were rough and sometimes irregular, special care was taken to select the best coupling transducer-material to improve the signal transmission by filling voids and irregularities at the interface. Silicone snug sheets were used as the coupling agent. This kind of coupling agent effectively contributes to a better transmissibility of the ultrasonic signal, avoiding possible penetration into the porous material and, consequently, preventing any unwanted interference with the material itself. 

The transit time of the propagation of the longitudinal ultrasonic wave from the transmitter to the receiver was measured in the direct transmission mode [59]. To improve the signal/noise ratio, and thus the data quality, a stack of five waveforms with the same travel path was carried out. The first arrival of the received longitudinal stacked signals was detected from the analysis of the waveforms recorded and displayed on the oscilloscope. Filtering was applied to further improve the signal/noise ratio, when necessary. In this case, linear tendency removal was sufficient. In a complex situation, such as the studied one, the automated arrival picking techniques cannot consistently detect first arrivals. Therefore, we handpicked the first arrivals of the longitudinal waves for the tomographic analysis. In fact, accurate first arrival picking is needed for tomographic data processing and directly influences the quality of the results.

The propagation longitudinal velocity (Vp) between the source and receiver transducers is expressed as the ratio of the source–receiver distance to the transit time. The Vp was used as an indirect parameter to assess the non-destructive detection of the textural variations, weakness, and weathering zones inside the materials [3,17].

#### 3D Ultrasonic Tomography

The 3D ultrasonic tomography volume was determined by positioning the source and receiver points along the perimeter of the investigated column shafts (circumscribed circle diameter: 1.49 m, on average). In each column, every station point was located with a 15 cm vertical spacing along parallel vertical profiles in such a way that they surrounded the investigated column shaft entirely and homogeneously and crossed the investigated volume in many directions. Each measurement point was used alternatively as a transmitter and a receiver. The locations of the measurement points, their coordinates in a fixed reference system, and the transmitter–receiver distance were precisely detected by 3D CRP and TLS models.

The ultrasonic waveforms recorded on each column shaft, which differed slightly in size, were, on average, 6560, but not all were processed by the inversion technique. In fact, during the quality control of the ultrasonic waveforms, only the first arrivals with shapes that looked undistorted were taken into account. 

The investigated volume was discretized into elements (voxels). The size of the voxels was determined by the number of source–receiver paths, and each voxel of the analyzed volume was crossed by multiple ray paths. This iterative procedure allowed to calculate the velocity of the longitudinal ultrasonic signal within each voxel. A tomographic inversion, starting from the position of the transmitters and receivers on a 3D Cartesian grid and the first break transit times between the source–receiver pair, was processed by considering curved rays and applying the well-known Simultaneous Iterative Reconstruction Technique (SIRT) algorithm that was introduced by Trampert and Leveque (1990) [60] to produce the 3D velocity distribution model in the investigated volume. The inversion problem assumes an initial velocity model and computes the travel time for each source/receiver path; then, the calculated and picked times are compared. The residuals are calculated, and a correction factor for the time discrepancy for each source–receiver path is applied to the velocity value of the cells contributing to the 3D model. The iterative process is repeated until the desired accuracy is obtained. 

In order to obtain a realistic initial velocity model as input for the SIRT to invert travel-time data and produce a reliable 3D representation of the ultrasonic longitudinal wave velocity distribution inside the columns, a methodology based on the cross-correlation function proposed by Fais and Casula (2010) [61] was applied [3,12,14]. In this study, the entire processing sequence was repeated for 20 iterations. This number of iterations was found to be appropriate to obtain the best representation of the 3D distribution of the longitudinal velocity within the investigated columns and represents a good compromise between the resolving power and the reliability of the velocity model.

The 3D rendering of the longitudinal velocity distribution inside the investigated volumes as a tomographic inversion result was represented by Voxler v.4.3.771 by Golden Software. This enabled efficient and accurate three-dimensional representation. This representation allowed to freely rotate the tomographic data volume, so that it could be examined from different orientations to provide an intuitive feel of the distribution of the elastic characteristics of the carbonate materials inside the columns. Furthermore, to facilitate the imaging of the longitudinal velocity distribution in the inner parts of the columns, the 3D volumes were sliced horizontally along their longitudinal axes. The selected horizontal slices were used to extract meaningful information from the 3D tomographic volumes. Therefore, based on the analysis of the horizontal slices, the distribution and evolution of the low-velocity zones associated with weakness zones or textural variations inside the investigate materials could be analyzed more easily. 

These 3D data volume visualizations can aid in conveying the results of the diagnostic analysis to other technicians for further applications (e.g., planning restoration). 

## 3. Results and Discussion

In this section, we present the results of the integrated application of the multiparametric techniques, e.g., CRP, TLS and 3D ultrasonic tomography, complemented with OM and SEM. It has been recognized that the geometrical anomalies, reflectivity maps, and 3D ultrasonic tomographies of the investigated columns are affected by the textural characteristics, nature, and distribution of porosity of the carbonate building materials as well as by the presence of defects of a different type and degree of weathering. Knowledge of the petrographical and petrophysical characteristics of the carbonate building materials allows a reliable analysis and interpretation of the results of the non-invasive techniques applied here.

### 3.1. Thin Section Analyses

**Figure 7:**
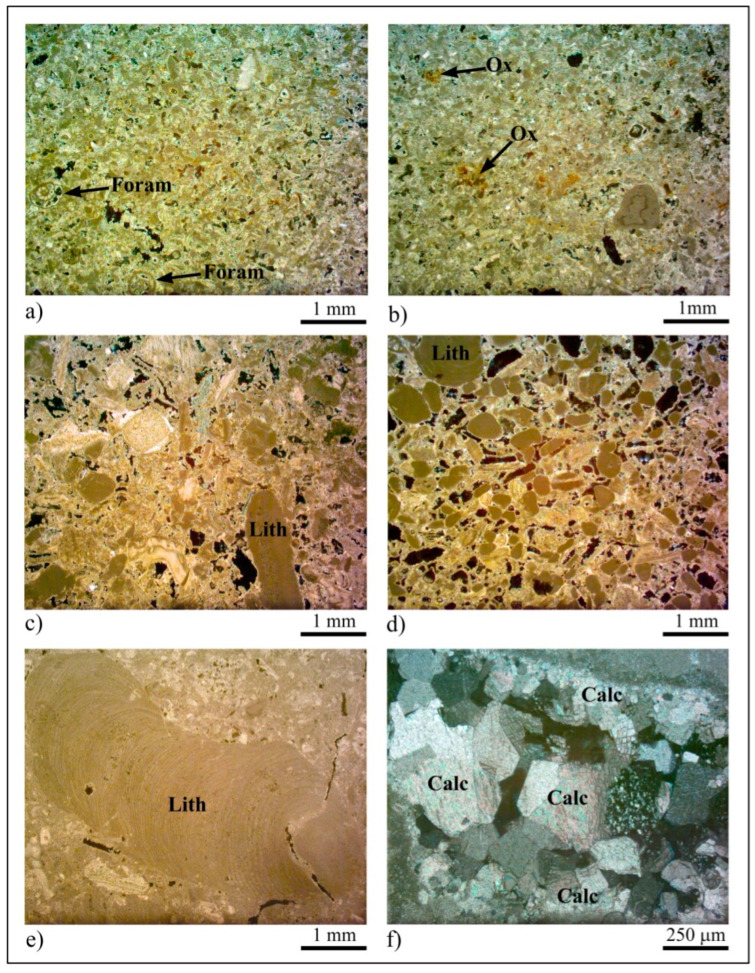
Textural characteristics of Calcari di Cagliari Auct. (**a**) Foraminifera (Foram) in mud-supported Pietra Cantone, OM plane polarized light; (**b**) iron oxide (Ox) specks in Pietra Cantone, OM plane polarized light; (**c**) bioclasts cemented by sparry calcite in Tramezzario. Lith (Lithothamnium algae), OM plane polarized light; (**d**) sector rich in Lithothamnium (Lith) algae fragments in Tramezzario, OM plane polarized light; (**e**) Lithothamnium (Lith) algae in Pietra Forte, OM plane polarized light; (**f**) Sparry calcite (Calc) crystals in the Pietra Forte cement, OM cross polarized light.

Based on thin section analyses and according to the Dunham classification (1962), Pietra Cantone may be classified as mudstone–wackestone. The allochems are made from well-sorted, sparse bioclasts with sizes ranging from 100 µm to 2.5 mm, mainly characterized by foraminifera supported by a micrite matrix (Figure 7a). Variable amounts of terrigenous minerals and iron oxides that give the rock its typical yellow color (Figure 7b) can be present in the matrix. Tramezzario can be defined as grainstone–packstone [62] characterized by bioclasts (Figure 7c) that are mostly made from well-sorted, sub-rounded algal fragments (Figure 7d) and remains of mollusks with sizes ranging from 500 µm to 4 mm, supported by sparry calcite cement. Pietra Forte can be classified as boundstone [62] mainly made from Lithotamnium algae (Figure 7e) well cemented by sparry calcite (Figure 7f).

Concerning the porosity of the investigated carbonate lithotypes, OM and SEM analyses allowed us to define the different grains–matrix or grains–cement relationships and the bioclast packing, highlighting the different types of porosity. The porosity detected in OM can be classified as mesoporosity [63,64], while the pores analyzed by SEM can be classified as microporosity [1,2].

**Figure 8:**
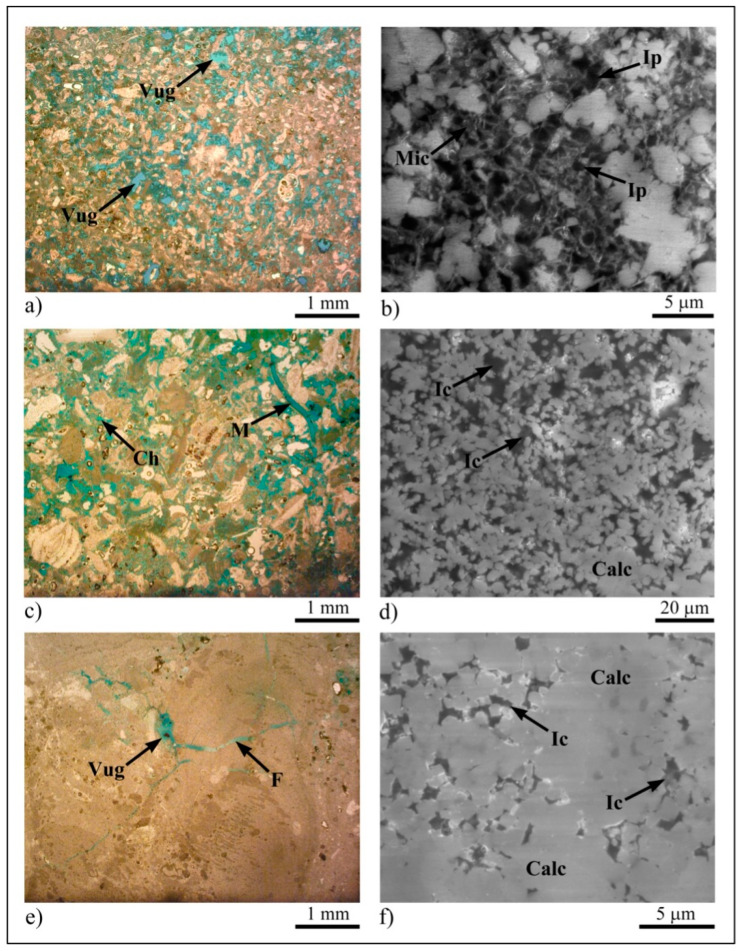
Porosity of Calcari di Cagliari Auct. (**a**) Vug porosity (Vug) in Pietra Cantone, OM plane polarized light; (**b**) interparticle porosity (Ip) in micrite mud (Mic) of Pietra Cantone, SEM backscattered-electron (BSE) image, EHT = 20 kV, WD = 10 mm; (**c**) moldic (M) and channel (Ch) porosities in Tramezzario, OM plane polarized light; (**d**) intercrystal (Ic) porosity in Tramezzario calcite (Calc) cement, SEM BSE image, EHT = 20 kV, WD = 10 mm; (**e**) Vug and fracture (F) porosities in Pietra Forte, OM plane polarized light; (**f**) intercrystal (Ic) porosity in Pietra Forte sparry calcite (Calc) cement, SEM BSE image, EHT = 20 kV, WD = 10 mm.

The porosity of Pietra Cantone can reach values of about 25%. The mesoporosity is characterized by primary interparticle pores that, due to the dissolution processes, can evolve into forms of secondary porosity, such as vug porosity (Figure 8a). The SEM analysis showed that Pietra Cantone is characterized mainly by a micropore network of well-connected interparticle pores with a mean size of 2 μm, concentrated in the micrite matrix (Figure 8b). The values of effective porosity measured by MIP on five representative samples of Pietra Cantone ranged from 25.35% to 38.71% with a mean value of 30.41 ± 4.47% (Table 1); these values are compatible with the high values of porosity determined by the OM and SEM analyses.

In Tramezzario, the porosity is 10% on average, but it can reach 20%, depending on the dissolution degree of the rock. The mesoporosity is of the secondary type [65] and is mainly of the channel, moldic (Figure 8c), intraparticle, and fracture types. These pores, except for the fractures, occur due to dissolution processes and appear to be well connected. In the SEM, the Tramezzario appeared to be made from single subhedral–anhedral calcite microcrystals (sizes 2–3 μm) or characterized by polyhedral aggregates of the same mineral. Intercrystal pores [65] with sizes between 4 μm and 5 μm (Figure 8d) represent the main microporosity of the Tramezzario. These pores, located within the sparry calcite cement, are generally interconnected (Figure 8d). The values of effective porosity measured by MIP on five representative samples of Tramezzario were generally high. They were within the range of 13.40–40.85% (Table 1) with a mean value of 27.02 ± 9.02%.

In Pietra Forte, the porosity is about 1–4% and is mainly of the secondary type [65], including intraparticle, vug and fracture pores (Figure 8e). However, pores of the primary type, such as growth frameworks due to the growth of Lithothamnium algae, are present in a few cases. Pietra Forte is exclusively made up of calcite, characterized by single subhedral microcrystals (sizes 2–3 μm) or polyhedral aggregates of calcite. In the SEM analysis, Pietra Forte appeared to be characterized by an intercrystal microporosity [65] with poorly interconnected pores with dimensions smaller than 4 μm (Figure 8f). According to OM and SEM, the results of the MIP analyses for five representative samples of Pietra Forte showed low effective porosity values ranging from 2.21% to 9.24% (Table 1) with a mean value of 6.62 ± 2.49%. Considering all aspects discussed above, we can point out that the intrinsic textural features of Pietra Cantone and Tramezzario, on which large amounts of the porosity and the pore types depend, strongly affect the degradation, especially when used as building materials. In fact, during the wet season when the humidity is greater, the water vapor penetrates into the pores, causing internal compressive forces that cause the rocks to expand in volume. During the dry season, when the moisture in the pores tends to leave the rock and the salts carried by marine aerosol, crystallize within the pores, the resulting internal tension forces cause stresses and a consequent variation in volume that leads to pulverization, detachment, and cracking in the material’s surface, as shown in Figure 3a,b. The textural features of Pietra Forte, on which the hardness of this rock depends, lead to its low porosity values. Therefore, Pietra Forte is less subjected to degradation phenomena. Notwithstanding this, even in this lithotype, in the case of high porosity, as occurs in the presence of karst cavities and/or discontinuities, degradation phenomena can also occur, and the material worsens significantly, as shown in Figure 3c. In fact, in these cases, the oxygen in the water that seeps into the fractures causes oxidation and, consequently, the formation of patinas of alteration that weaken the surface materials.

### 3.2. Close-Range Photogrammetry and Terrestrial Laser Scanner

**Figure 9:**
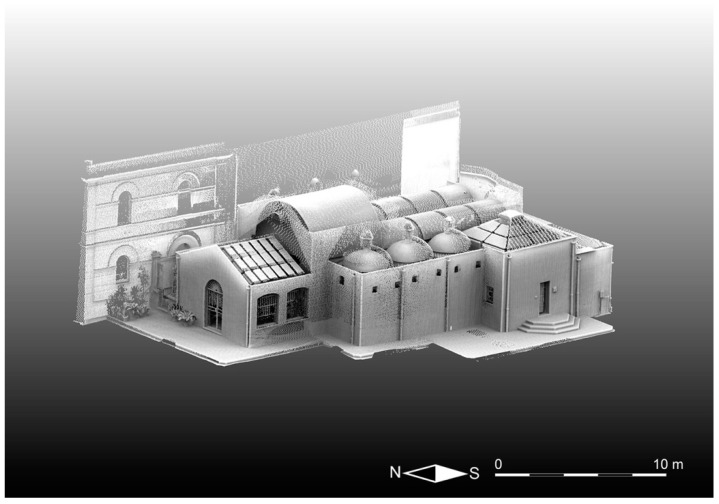
Unified, filtered, registered, unstructured point cloud texturized with the intensity that represents the high-resolution 3D model of the church of Saints Lorenzo and Pancrazio computed after TLS data processing.

**Figure 10:**
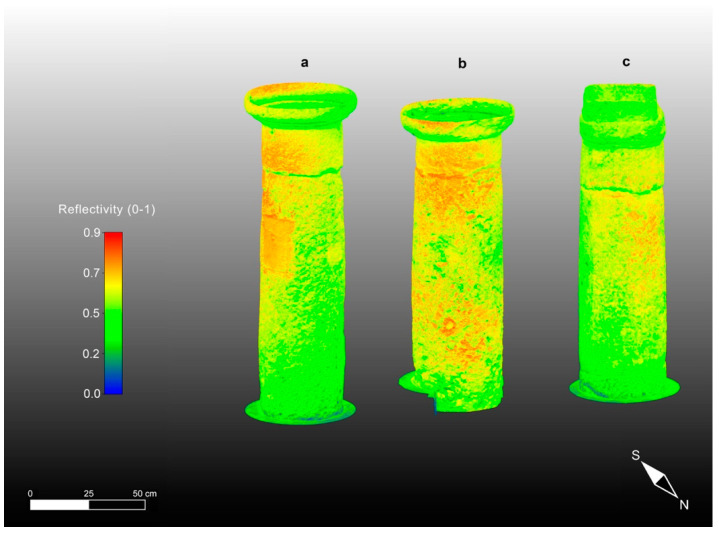
High-resolution 3D models of the studied columns from the Saints Lorenzo and Pancrazio church texturized with the intensity obtained after TLS data processing: (**a**) Column 1; (**b**) Column 2; (**c**) Column 3.

Notably, the first results obtained with the TLS methodology were the high-resolution 3D models (i.e., aggregated, unstructured point clouds) texturized with an intensity parameter that can be considered practically proportional to the reflecting power of the targeted surface materials and comparable with the velocity patterns of the ultrasonic tomography. The first aggregated, filtered, and registered cloud represents the entire Saints Lorenzo and Pancrazio church building, containing approximately 1.3 billion points (Figure 9). Subsequently, using TLS data, we formed 3D models of the three studied columns, each one containing approximately tens of millions of points, as presented in Figure 10, and immediately comparable with the corresponding models obtained with the application of CRP. 

**Figure 11:**
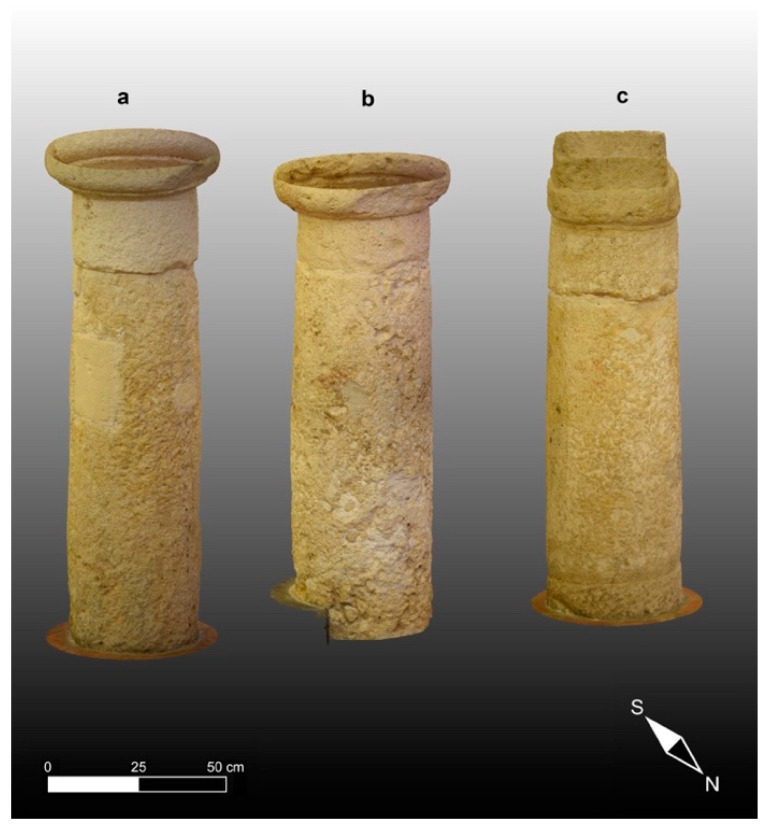
High-resolution 3D models of the three studied columns computed with CRP photogrammetry data processing and texturized with the natural colors of the high-quality 2D images: (**a**) Column 1; (**b**) Column 2; (**c**) Column 3.

Regarding the CRP methodology, we computed similar high-resolution 3D models texturized with the natural colors of the building materials that make up the studied columns and subsequently calibrated adjusting scales and coordinates for direct comparison with the corresponding TLS models; the latter models are presented in Figure 11.

**Figure 12:**
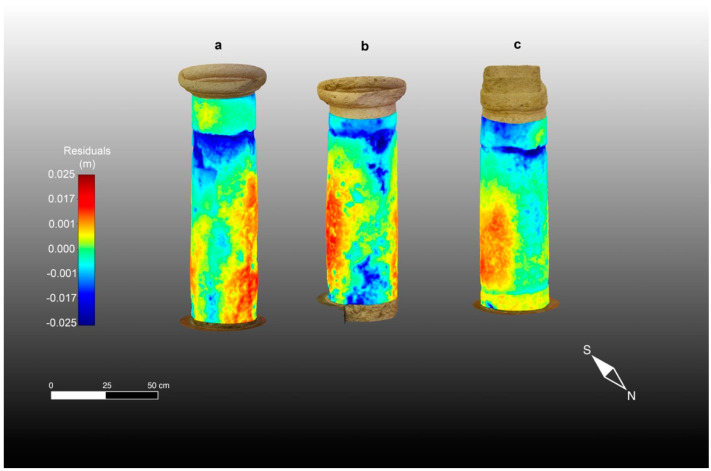
Geometrical Anomalies of the three studied columns representing the residuals compared with a fitted cylindric geometry adopted as reference superimposed onto the photogrammetric high-resolution 3D models: (**a**) column 1; (**b**) column 2; (**c**) column 3.

**Figure 13:**
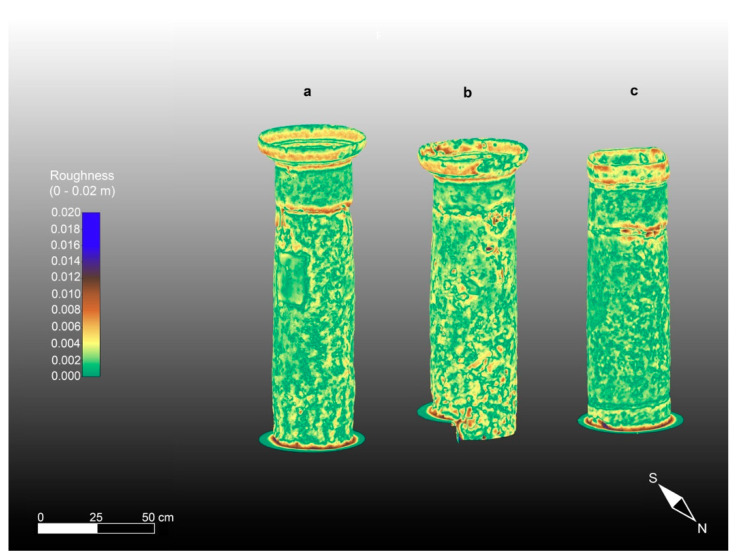
Roughness patterns of the three studied columns superimposed onto the 3D high resolution CRP models: (**a**) Column 1; (**b**) Column 2; (**c**) Column 3.

The second product of the synergistic application of TLS and CRP is the morphological anomalies of the studied columns, which were computed from the calibrated CRP 3D models, as described above, using the respective geometrical residuals and roughness patterns. These morphological anomalies are presented in Figure 12 and Figure 13, respectively.

The three aggregated point clouds representing the three studied columns contained exactly 12, 21, and 33 million points, respectively. They had a maximum available density of more than 1 point every 0.1 mm. The following general considerations can be derived from these high-resolution 3D models. Column 1 is high and tight with a mean ray of about 0.226 m, column 2 is lower and wider with a mean ray of about 0.246 m, and column 3 is in the middle with a ray of 0.239 m. 

As can be seen by looking at the three high-resolution 3D digital models derived from CRP complemented by the application of TLS methodologies, columns 2 and 3, but especially 2, appear to be more deformed along the vertical axis than column 1. This could be due to the years-long action of normal stress from the load of the round arches that partially rest on the squat ancient columns. Moreover, as seen from the macroscopical analyses performed in advance, evidence of restructuring operations over the years is visible along the shallow parts of the columns, which are mainly characterized by applications of mortar reinforced with stone fragments and inserts of stone material (Pietra Cantone) of rectangular shape.

Regarding the roughness (see Figure 13). column 2 appears to be the most damaged. As a matter of fact, the roughness pattern shows greater residuals varying in the 0–2 cm range, particularly at the base of the column shaft and in its upper part, just below the capital, and in an area close to the joint. Column 3 is less damaged than column 1. They both show a pattern of geometrical and roughness anomalies with little variation (0–1.5 cm). In fact, looking at Figure 13, the roughness patterns of the three studied columns can be seen to range between low values (0–2 mm), represented by green hues, and higher anomalies (about 2 cm or greater in the case of column 2), represented by red and blue hues. This condition is also confirmed by the TLS intensity patterns and macroscopical analyses and is mainly due to the natural macropores caused by the dissolution phenomena of the calcite. In particular, this occurs in the areas of the artifacts in which the concentration of fossils is greater. In the resulting morphological maps, regarding the geometrical anomalies presented in Figure 12 corresponding to the studied columns, it is noteworthy that the color scale used for the maps ranges between a negative residual of −2.5 cm, presented in dark blue, and a positive residual of the same amount (2.5 cm), presented in bright red. In particular, the dark blue areas are evident in the sectors of the columns where small cavities and voids are presented as blue and green spots, while the central parts of the maps show undamaged material (positive residuals) in red to yellow (see Figure 12). It is clear that the morphological maps (roughness and geometrical) have also been affected by the shape of the columns, which is wider at the base of the shaft and narrows at the top near the junction with the capital. In any case, undamaged parts can be seen to alternate with zones characterized by degraded material compared with the regular geometry of the fitted cylinders adopted as a reference. In the capitals, the negative anomalies are mainly caused by the detachment of some material due to physical causes. Conversely, shallow chemical alteration processes, such as oxidation, occur mainly at the base of the shaft, causing a reddish coloring of natural RGB in this area of the artifacts.

### 3.3. Ultrasonic Tomography

The 3D ultrasonic tomography of the three analyzed columns produced high-resolution images of the distribution of the longitudinal velocities in the investigated volumes. As a first result of the ultrasonic evaluation, the average velocity was detected for each column. The average velocities were 2410 m/s in column 1, 1600 m/s in column 2, and 3400 m/s in column 3. As already recognized in previous works, and considering previous ultrasonic laboratory tests [5,12,25] on the same carbonate building materials (Pietra Forte), the ultrasonic wave velocity is about 5000–6000 m/s for sound in Pietra Forte, but it can decrease to values of 1000–2000 m/s when damaged. It is known that the greater the propagation velocity of the longitudinal wave is, the better the quality of the stone materials is.

**Figure 14:**
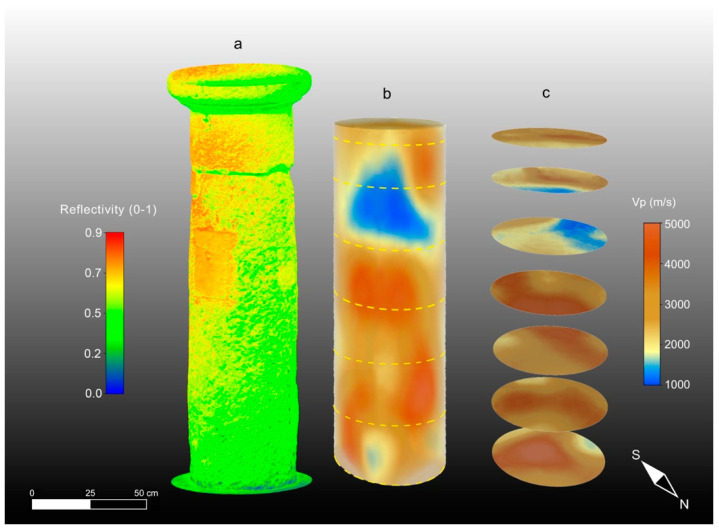
High-resolution 3D models of column 1. (**a**) TLS reflectivity model; (**b**) ultrasonic tomography; (**c**) tomographic slices.

**Figure 15:**
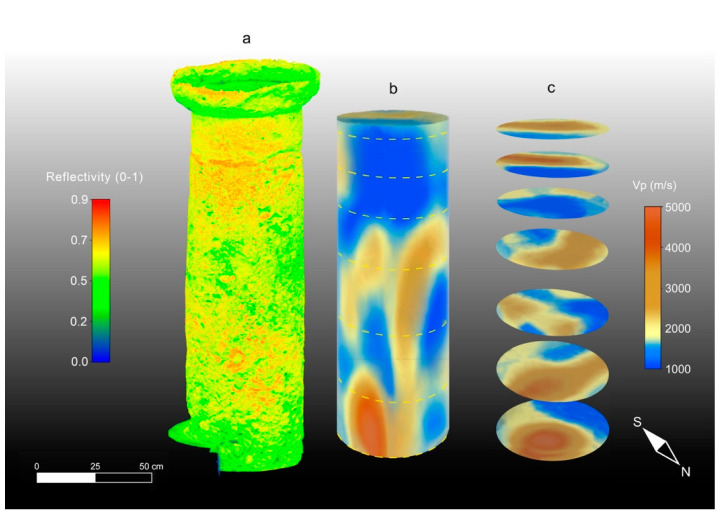
High-resolution 3D models of column 2. (**a**) TLS reflectivity model; (**b**) ultrasonic tomography; (**c**) tomographic slices.

**Figure 16:**
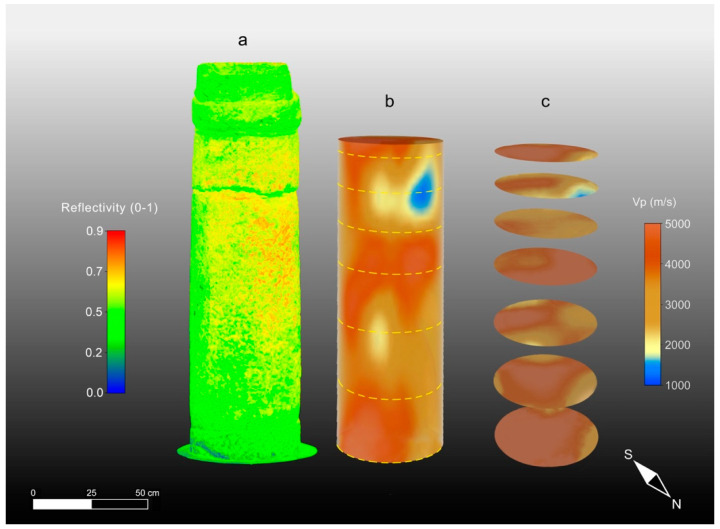
High-resolution 3D models of column 3. (**a**) TLS reflectivity model; (**b**) ultrasonic tomography; (**c**) tomographic slices.

The results of the 3D ultrasonic tomography with the P-wave velocity distribution on the three columns are presented together with the 3D TLS reflectivity models in Figure 14a–c, Figure 15a–c and Figure 16a–c. An analysis of the 3D ultrasonic tomographic volumes showed a high velocity variability ranging from 1000 m/s to 5000 m/s.

From the analysis of the 3D tomography on each column, it is clear that column 2 (Figure 15b,c) is characterized by a higher variability in the longitudinal velocity value compared with the other two columns (1 and 3) (Figure 14b,c and Figure 16b,c). In column 2, the elastic characteristics of the Pietra Forte are generally poor for different reasons: (1) deformation along the longitudinal axis of the column shaft, as observed in the high-resolution 3D digital models derived with the CRP and TLS methodologies; (2) the presence of macropores (e.g., small karst cavities) and microfractures that result in a significant decrease in the longitudinal velocity; and (3) textural variations of the carbonate material, especially the different types of porosity, and the presence of discontinuities among the bioclasts [16,17]. In particular, points 2 and 3 increase the roughness of the shallow part of the column shaft which, in turn, affects the TLS reflectivity, as can be observed in Figure 15a. The comparison between the 3D TLS reflectivity model and the 3D ultrasonic tomography (Figure 15a,b) clearly highlights that the low-velocity sectors (shown in blue) are almost in correspondence with the high-reflectivity ones (red), where the roughness caused by the degradation of the shallow materials is greater. This is especially evident in the upper part of the column shaft, where reflectivity values in the range of 0.7–0.9 correspond to velocity values in the range of 1000–1800 m/s, which is typical of quite intensively altered Pietra Forte. In this particular case, the longitudinal velocity decreases, probably due to a worsening of the mechanical properties of the material, related to a deformation, as is well-evidenced by the high-resolution 3D CRP and TLS models, and the presence of a joint. This deformation is probably due to an inappropriate load distribution on the supporting structure.

Horizontal slices through the longitudinal development of the 3D tomographic volume (Figure 15c) provided high-resolution images of the inside column, allowing us to better understand the three-dimensional development of the low-velocity zones (colored blue) associated with Pietra Forte degradation as well as to detect their geometry and size.

Taking into account the results of a previous laboratory test on the same materials [25] and the results of the petrographical analyses carried out in this study, the low-velocity zones inside the column could be also related to the presence of small karst cavities or discontinuities caused by the dissolution phenomena of calcite.

**Figure 17:**
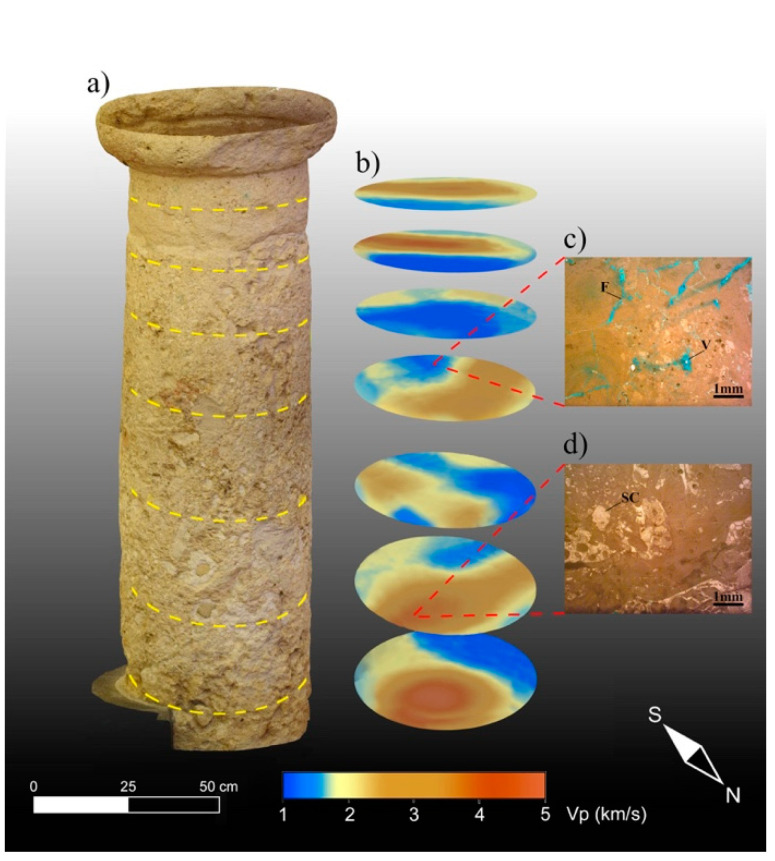
Integrated analysis of the ultrasonic tomography and thin sections for column 2. (**a**) CRP model; (**b**) tomographic slices; (**c**) thin section of the Pietra Forte in a sound sector of the shaft; (**d**) thin section of the Pietra Forte in a degraded sector of the shaft. F = fracture, V = vug, SC = sparry calcite cement.

In sectors with higher velocities (colored brown) corresponding to lower TLS reflectivity values (Figure 15a,b), the elastic characteristics were improved due to a greater compactness and a higher degree of cementation, which reduced the porosity and discontinuities inside the material. In Figure 17a–d, a possible correlation between the velocity distribution inside column 2 and the textural characteristics of the investigated carbonate materials is presented, together with the CRP model. 

The 3D ultrasonic tomographies and the horizontal tomographic slices of columns 1 and 3 (Figure 14b,c and Figure 16b,c) show that only small areas close to junctions under the capitals are characterized by low-velocity values (blue) that can be correlated with higher-intensity values (red) of the TLS reflectivity (Figure 14a and Figure 16a). Column 3 shows quite a homogeneous ultrasonic velocity distribution, indicating the better elastic conditions of the building material compared to column 1 and especially column 2. Based on the above, and also considering the results obtained with TLS and CRP methodologies, it is evident that the amount of damaged material compared to unaltered material is about 60% in column 2. Conversely, it is about 20% in column 1 and 15% in column 3.

## 4. Conclusions

The synergistic application of different ND techniques is of paramount importance for the study of ancient monuments and artefacts, such as the columns of the Saints Lorenzo and Pancrazio church, which were the main object of this study. The characteristics of the building stone materials used in the past to assemble monuments must be carefully inspected from petrographical/petrophysical, optical, and metrical aspects to all elasto-mechanical properties by analyzing the propagation velocity of the acoustic signal through the materials. All datasets can complement each other and overcome their limits, thus giving a complete high-resolution 3D image of the studied artefacts so as to produce guidelines for their conservation and restoration processes.

In this paper, we therefore applied an integrated procedure aimed at obtaining the most complete characterization of the stone materials and an understanding of their conservation states by analyzing the processes that are at the base of their degradation.

Moreover, an accurate study on the textural characteristics of the investigated carbonate materials was carried out by OM and SEM analyses. In fact, understanding the main petrographical features of these materials is indispensable, due to the heterogeneity and complexity of their textural components and considering the relationships with their elastic properties and other physical parameters.

After the synergistic application of the CRP, TLS, and ultrasonic tomography methods, we were able to locate and quantify the altered parts and weakness zones of the materials making up the three studied columns of the Church of Saints Lorenzo and Pancrazio at both the surface and deeper into the materials. Based on the above, it is evident that the amount of damaged material compared to unaltered material is about 60% in column 2. Conversely, it is about 20% in column 1 and 15% in column 3. Thanks to the 3D multiparametric high-resolution models of the studied columns, it was possible to produce images of the external and internal parts of the three ancient columns, thus preserving the memory of the shapes and implementing a system that can monitor the evolution of degradation over time by repeatable tests.

## Figures and Tables

**Table 1:** Effective porosity of *Calcari di Cagliari* determined by MIP analyses.

Lithofacies of Calcari di Cagliari	Samples	Porosity (%)	
			Mean Values ± Standard Deviation
Pietra Cantone	PC1	25.35	30.41 ± 4.47
	PC2	38.71	
	PC3	28.86	
	PC4	30.41	
	PC5	28.72	
Tramezzario	TR1	40.85	27.02 ± 9.02
	TR2	13.40	
	TR3	26.61	
	TR4	23.25	
	TR5	31.00	
Pietra Forte	PF1	6.26	6.62 ± 2.49
	PF2	9.24	
	PF3	8.74	
	PF4	6.66	
	PF5	2.21	

## Data Availability

Available upon request to the corresponding author.
